# Utilization of personal protective equipment and its key factors among WA oil factory workers in Debre Markos town, Ethiopia

**DOI:** 10.3389/fpubh.2025.1529436

**Published:** 2025-05-30

**Authors:** Abraham Teym, Tirsit Ketsela Zeleke

**Affiliations:** ^1^Department of Environmental Health, College of Health Sciences, Debre Markos University, Debre Markos, Ethiopia; ^2^Department of Pharmacy, College of Health Sciences, Debre Markos University, Debre Markos, Ethiopia

**Keywords:** utilization, personal protective equipment, edible oil factory, factory worker, Ethiopia

## Abstract

**Background:**

Edible oil manufacturing is a labor-intensive sector with significant technological demands, where employees face various occupational hazards. The use of personal protective equipment (PPE) is not only a legal obligation but also a key measure for safeguarding workers against job-related injuries and health risks. Despite these challenges, this industry often remains under-researched and overlooked.

**Objective:**

To assess utilization of personal protective equipment and its key factors among workers in the WA edible oil factory in Debre Markos town, Ethiopia, in 2024.

**Methods:**

A cross-sectional study was conducted among employees of the WA Edible Oil Factory in Debre Markos. Using a simple random sampling method, 387 workers were selected to participate. Data were collected through an interviewer-administered structured questionnaire, focusing on the use of protective equipment, as well as socio-demographic, work-related, environmental, and organizational characteristics. The data were analyzed using SPSS version 26. Logistic regression analysis was employed to identify factors influencing the use of protective equipment, with the strength of associations expressed as odds ratios at a 95% confidence level.

**Results:**

Out of the total workforce, 214 individuals (55.3%) reported using personal protective equipment while on duty. The study identified several significant factors influencing personal protective equipment utilization, including receiving safety training, having access to protective equipment, regular occupational health and safety inspections, the presence of workplace safety protocols, having three or more years of work experience, and abstaining from alcohol consumption and smoking.

**Conclusion:**

The utilization level of personal protective equipment among workers at the WA edible oil factory was found to be moderate when compared to findings from other developing countries. Key factors influencing personal protective equipment usage included access to safety training, availability of protective gear, workplace supervision, the presence of safety protocols, work experience, and lifestyle behaviors such as alcohol and tobacco use. To improve personal protective equipment utilization, it is recommended to strengthen workplace supervision, offer comprehensive safety training, and ensure the consistent availability of safety guidelines.

## Introduction

### Background

The use of personal protective equipment (PPE) is a key measure for minimizing occupational injuries and illnesses caused by exposure to various workplace hazards. PPE is specifically designed to safeguard workers from serious harm resulting from contact with chemical, physical, electrical, radiological, mechanical, or other hazardous agents present in the work environment. Common types of PPE include gloves, safety goggles, protective footwear, earplugs or earmuffs, hard hats, respirators, vests, and full-body suits (1). It is a significant determining factor between an accident and safety in the working environment. Evidence suggests that wearing the correct personal protection at all times is extremely important in reducing accidents and should be given high priority (2).

Edible oil manufacturing plants play a vital role in enhancing food self-sufficiency, supporting the national economy, and promoting sustainable development. These facilities not only improve public nutrition but also present valuable opportunities for both local and foreign investment (3). However, like many other industrial processes, edible oil manufacturing poses considerable risks to worker health and safety, as well as potential negative impacts on the surrounding environment (4). An accident that occurs in the industrial context, will pose a risk that harms people, property, and the environment.

Occupational injuries pose major public health and developmental problems; which result in serious health, social, and economic consequences on workers and their employers (5). According to an International Labor Organization (ILO) report, there are 317 million workplace accidents each year, including falls, slips, and machinery-related incidents. The 6,300 deaths may result from severe accidents, workplace diseases like lung diseases, or mental health issues caused by work-related stress or environmental hazards (6). Employees must understand when is necessary to use, what equipment is required, how to use or wear, how to care, how to know when the equipment has reached the end of its useful life and how to dispose of PPE (7–9). The prevalence of occupational injury is high which is by low PPE usage (10). Globally, 34% of occupational accidents were resulting from the lack of use of PPE available at workplace at the time of the accident (11). In addition, 13% of work-related accidents result from the inappropriate use of PPE (11).

The use of PPE among factory workers remains low and is significantly linked to the availability and quality of safety training. This is important because workplace accidents are frequently associated with inadequate safety practices and insufficient PPE use (12). Studies consistently report that PPE utilization is particularly limited across Africa (13–15). As a result, many workers are left vulnerable to numerous physical, chemical, and accidental hazards due to the lack of proper protective measures (16). About half of Ethiopia's workforce suffer from occupational injuries, and not wearing PPE was a major factor (17).

Multiple factors have been associated with the low utilization of PPE among factory workers in Ethiopia. Key barriers include limited awareness, insufficient training, lack of proper safety orientation, inconsistent supervision, and inadequate supply of PPE materials, all of which significantly influence workers' adherence to occupational safety measures (18). Edible oil factories are labor-intensive and operate using complex technologies, which expose workers to a range of occupational hazards, including chemical agents, dust, and mechanical injuries particularly during the extraction and refining processes. Despite these risks, occupational safety practices in such industries remain largely under prioritized in Ethiopia, with limited research addressing the issue. Therefore, this study aimed to assess the level of PPE utilization and examine the factors influencing its use among workers at the WA Edible Oil Factory in Debre Markos Town, Northwest Ethiopia.

## Materials and methods

### Study area

The study was conducted at the WA Edible Oil Factory in Debre Markos Town, East Gojjam, Amhara Region, Northwest Ethiopia. The area is located 300 kilometers from Addis Ababa, the capital of Ethiopia, and 265 kilometers from Bahir Dar, the capital of the Amhara Region. The WA Edible Oil factory in Debre Markos town was constructed by well-known Ethiopian investor Worku Aytenew and was inaugurated in 2021. This factory can process over 1.5 million litters daily. Oil is extracted from Niger, peanut, soy and sesame seeds (19).

### Study design and period

An institutional-based cross-sectional study was carried out between March and April 2024.

### Source population

All WA edible oil factory workers in Debre Markos town were considered as a source population.

### Study population

All workers who are directly involved in the process of production in WA edible oil factory were included until the required sample size was achieved. The factory workers who were selected as a study subjects were considered as a study population.

#### Inclusion criteria

Workers in the selected industry who receive salaries or wages and have been on the payroll for at least 6 months or more before the study period were eligible to participate.

#### Exclusion criteria

Workers who were absent due to illness or on sick leave during the study period were excluded.

### Sample size determination

The required sample size for the study is determined using single population proportion formula according to the available literature taking the prevalence of PPE actual use as 38 (18).


$$\begin{array}{lll} \left(n\right) & = & \frac{{\left(Z\alpha /2\right)}^{2}P\left(1-P\right)}{{\left(d\right)}^{2}}\\ \left(n\right) & = & \frac{{\left(1.96\right)}^{2}0.0.38\left(1-0.38\right)}{{\left(0.05\right)}^{2}}=\;362 \end{array}$$

The sample size (n) was calculated using the following parameters: *z* = 1.96 for a 95% confidence interval, *P* = 38% (18) for the PPE utilization rate, and 1—P = 0.59 for the complementary probability. The margin of error (d) was set at 0.05. After accounting for a 10% non-response rate (18), the final sample size required to ensure representative data was determined to be 402 factory workers.

### Sampling technique

A stratified sampling method followed by simple random sampling was employed to ensure representativeness and reduce sampling bias in the study. Initially, the manufacturing workforce was stratified into six distinct departments based on their job functions: seed preparation, oil extraction, refining, quality control, packaging and storage, and maintenance and engineering. This stratification allowed the researchers to account for potential differences in exposure risks and PPE usage across departments. After stratification, the total sample size of 402 participants was proportionally allocated to each department according to the number of workers in that category, ensuring that departments with more employees contributed a correspondingly larger share of the sample. Finally, within each department, individual participants were selected using a simple random sampling technique from the factory's employee registry, giving each eligible worker an equal chance of being included in the study.

### Study variables

In this study, the utilization of PPE was treated as the primary outcome variable. A range of independent variables was assessed to determine their association with PPE use. These included socio-demographic characteristics such as age, sex, religion, educational level, marital status, type of employment, monthly income, and years of work experience. Behavioral factors considered included alcohol consumption, cigarette smoking, khat chewing, and job satisfaction. Individual-level factors encompassed knowledge of workplace hazards and awareness about the purpose and use of PPE. Additionally, work-related factors such as job role, employment status, duration of employment, availability of PPE, participation in safety training or orientation, presence of workplace supervision, work shifts and rotation schedules, lighting conditions, and ventilation were also examined as potential predictors of PPE utilization.

### Data collection tool and procedure

Data on socio-demographic, behavioral, individual, and work-related variables were collected through an interviewer-administered structured questionnaire. This tool was developed based on an extensive review of relevant literature (2, 14, 18, 20–22). The questionnaire was organized into four main sections: part I addressed socio-demographic characteristics, consisting of 8 items; Part II focused on behavioral factors, with 4 items; Part III assessed individual-level characteristics through 2 items; and Part IV explored work-related factors using 12 items. To ensure linguistic and conceptual consistency, the original English version of the questionnaire was translated into Amharic and then back-translated into English by independent language experts. A pilot test was conducted with 16 employees from the nearby Grace Biscuit Factory in Debre Markos town to evaluate the instrument's clarity and reliability. The Cronbach's alpha for the overall questionnaire was found to be 0.82, indicating acceptable internal consistency (values above 0.60 are considered acceptable) (23). Data collection was carried out by four Environmental Health professionals who received prior training specific to the study objectives and tools.

### Data management and statistical analysis

Data cleaning was conducted to ensure accuracy, completeness, consistency, and the absence of missing values or variables. The cleaned data were manually coded, entered into EpiData version 4.2.0.0, and then exported to SPSS version 26 for further analysis. Descriptive statistics, including means, standard deviations, frequencies, and proportions, were used to summarizeboth dependent and independent variables and describe the study population. To assess the suitability of the logistic regression model, the Hosmer–Lemeshow goodness-of-fit test was applied, yielding a *p*-value of 0.61, which indicates that the model fits the data well (*p* > 0.05). Multicollinearity among independent variables was evaluated using the Variance Inflation Factor (VIF), and no variable exceeded the threshold of 10, suggesting no multicollinearity concerns.

Both bivariate and multivariable logistic regression analyses were performed to identify factors significantly associated with PPE utilization. Variables with a *p* < 0.25 in the bivariate analysis were included in the multivariable model. The strength of association was determined using adjusted odds ratios (AORs) with 95% confidence intervals (CIs). Statistical significance was declared at a *p*-value of < 0.05.

### Operational definitions

#### Utilization of PPE

Use of all the necessary worker-specialized clothing or equipment by workers for protection against health and safety hazards in the workplace (22). Workers were classified as *those who used PPE* when they were observed wearing of all the PPE that were necessary to be worn during work in a particular working section. The necessarily worn PPE were: (1) a respirator, gloves, eye protector, boot shoes, overall, ear plugs and mask at spinning section, (2) respirator, gloves, eye protector, boot shoes, ear plugs and overall at weaving section, (3) respirator, gloves, mask, ear plugs, boot shoes and overall at finishing section, (4) respirator, gloves, boot shoes, eye protector, overall, reflector, mask and helmet at engineering section, and (5) gloves, boot shoes, mask and overall at garmenting section.

## Results

### Socio-demographic characteristics of the respondents

This study involved 402 factory workers, yielding a response rate of 96.3% (*n* = 387). The majority of participants were male, accounting for 260 respondents (67.18%). Most respondents were between 25 and 31 years old, representing 213 participants (55.04%). Regarding employment status, 374 respondents (96.64%) held permanent positions. The mean age of the participants was 28.59 years [standard deviation (SD) = 5.87], with ages ranging from 18 to 54 years. A large proportion of the respondents 360 individuals (93.02%) identified as followers of the Orthodox Christian faith. Concerning marital status, 198 (51.16%) were single. Educationally, 178 participants (45.98%) had completed college or higher education. Additionally, 40% of the respondents reported having between 1 and 3 years of work experience. The average monthly income was 6,372 Ethiopian Birr (ETB), with a standard deviation of 1,943 ETB (Table 1).

**Table 1:** Socio-demography characteristics of the respondent among WA oil factory workers, Debre Markos, Ethiopia, 2024.

**Variable**	**Category**	**Frequency**	**Percent (%)**
Sex	Male	260	67.18
	Female	127	32.82
Age (years)	18–24	85	21.96
	25–31	213	55.04
	32–38	65	16.79
	39–45	19	4.90
	>45	5	1.31
Religion	Orthodox	360	93.02
	Muslim	18	4.65
	Protestant	9	2.33
Marital status	Married	173	44.70
	Single	198	51.16
	Divorced	6	1.55
	Widowed	4	0.10
	Separated	6	1.55
Educational status	Unable to read and write	3	0.07
	Read and write	19	4.90
	Primary school (1–8)	85	21.96
	Secondary school (9–12)	102	26.35
	Degree or higher	178	45.98
Employment pattern	Permanent	374	96.64
	Temporary	13	3.36
Monthly income (ETB)	1,500–3,400	17	4.39
	3,500–5,400	125	32.29
	5,500–7,400	120	31.10
	7,500–9,400	89	22.99
	≥9,500	36	9.30
Type of work	Mechanic	92	23.77
	Welder	67	17.31
	Electrician	24	6.20
	Painter	16	4.13
	Plumber	10	2.58
	Carpenter	24	6.20
	Machinist	28	7.23
	Operator	56	14.47
	Loader/Off loader	52	13.43
	Cleaner	18	4.68
Work experience (years)	< 1	135	34.88
	1–3	157	40.56
	>3	95	24.56

### Utilization of PPE

**Figure 1:**
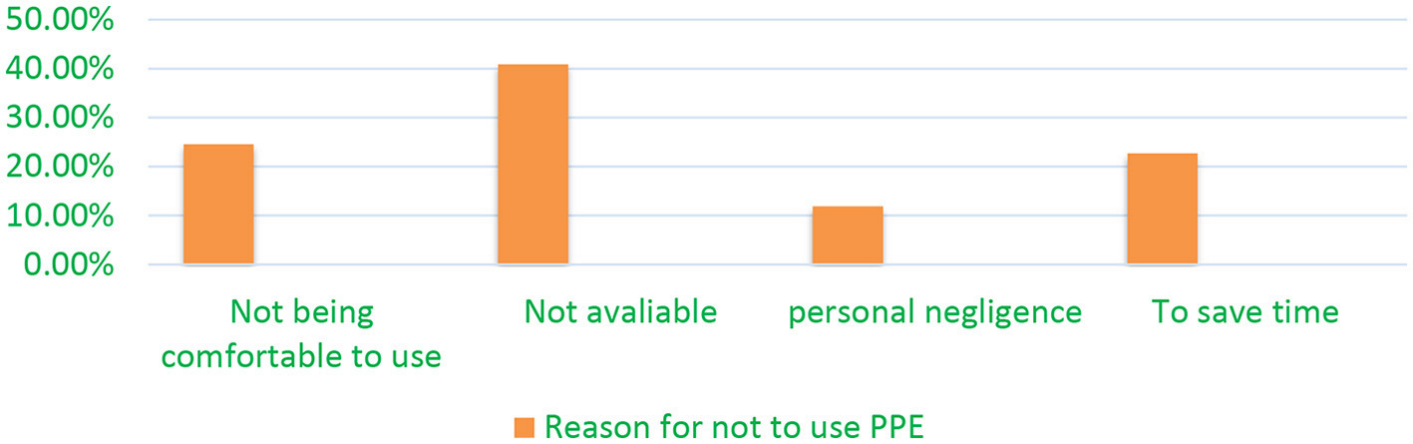
Reasons of the respondents for not to utilize the necessary PPE during work among WA oil factory workers, Debre Markos town, Ethiopia, 2024.

A total of 214 participants (55.29%) reported using all the required PPE during working hours, while 173 (44.71%) did not consistently use all necessary PPE. The four most commonly cited reasons for not using PPE were lack of availability, discomfort during use, the desire to save time, and personal negligence (Figure 1).

The most frequently used PPE were gloves (95.6%), overalls (90.18%), boots (100%), and masks (81.13%). In contrast, usage of earplugs (10.08%), goggles (19.45%), face shields/safety glasses (19.60%), and reflective vests (20.26%) was notably low. Helmet use was reported by 72.35% of participants (Table 2).

**Table 2:** Type and level of PPE utilization by WA oil factory workers, Debre Markos town, Ethiopia, 2024.

**Type of PPE**	**Use**	**Frequency**	**Percent (%)**
Glove	Yes	370	95.60
	No	17	4.40
Ear plug	Yes	39	10.08
	No	348	89.92
Mask	Yes	314	81.13
	No	73	18.87
Helmet	Yes	280	72.35
	No	107	27.65
Overall	Yes	349	90.18
	No	38	9.82
Goggles	Yes	43	19.45
	No	178	80.55
	Subtotal	221	100.00
Boots/shoes	Yes	295	100.00
	No	0	0.00
Face shield/Safety glass	Yes	20	19.60
	No	82	80.03
	Subtotal	102	100.00
Reflective vest	Yes	31	20.26
	No	122	79.73
	Subtotal	153	100.00

### Behavioral characteristics of respondents

The majority of participants reported not smoking (96.89%) and not chewing khat (97.41%). However, 43.67% reported alcohol use. Most workers (95.1%) expressed satisfaction with their jobs (Table 3).

**Table 3:** Behavioral characteristics of WA oil factory workers in Debre Markos town, Ethiopia, 2024.

**Category**	**Response**	**Frequency**	**Percent (%)**
Smoke cigarette	No	375	96.89
	Yes	12	3.11
Alcohol use	No	218	56.33
	Yes	169	43.67
Chew khat	No	377	97.41
	Yes	10	2.59
Job satisfaction	No	19	4.90
	Yes	368	95.1

### Environmental and organizational conditions

Nearly all respondents (98%) indicated that the factory provides PPE. A significant majority, 355 (91.73%), perceived their workplace as risky, and 327 (84.49%) reported receiving safety training related to new employment, equipment, or work processes. While 324 participants (83.73%) had received on-the-job PPE training, only 68 (17.57%) received PPE training upon first joining the job. Most workers (97.33%) observed that their co-workers use PPE, and 77.78% reported being encouraged by colleagues to do the same. Additionally, 62.01% noted the presence of regular health and safety supervision, and 66.67% believed they may be exposed to injuries or harmful substances at work. However, only 47.29% indicated the availability of safety guidelines in the workplace (Table 4).

**Table 4:** Working environment and organization conditions in WA edible oil, Debre Markos town, Ethiopia, 2024.

**Category**	**Response**	**Frequency**	**Percent (%)**
Training on any type of PPE issues when first engaged in this job	Yes	68	17.57
	No	319	82.43
Job training on any type of PPE	No	63	16.27
	Yes	324	83.73
The perceived workplace is a risk	No	32	8.27
	Yes	355	91.73
May be exposed to injuries or harmful substances at work	No	129	33.33
	Yes	258	66.67
Fellow workers use PPEs when they are working	No	8	2.67
	Yes	379	97.33
Co-workers encourage you to use PPEs	No	86	22.22
	Yes	301	77.78
Work-related injury	No	365	94.31
	Yes	21	5.69
Regular Health and Safety Supervision	Yes	240	62.01
	No	147	37.99
Safety training in connection with new	No	60	15.51
employment, equipment, or Work Process	Yes	327	84.49
Safety orientation before starting the job	No	145	37.46
	Yes	242	62.54
Safety Guidelines in the Workplace	No	204	52.71
	Yes	183	47.29
Work shift	No	40	10.33
	Yes	347	89.67
Work rotation	No	345	89.14
	Yes	42	10.86

### Factors that affect the utilization of PPE

The multivariable logistic regression analysis revealed that workers who received safety training were 4.77 times more likely to utilize PPE compared to those who did not [AOR = 4.77; 95% CI: 2.85–8.21]. Similarly, the odds of PPE utilization were significantly higher among workers with access to and availability of PPE [AOR = 4.92; 95% CI: 2.35–7.12]. The presence of health and safety supervision [AOR = 2.81; 95% CI: 1.90–3.99] and the availability of safety guidelines in the workplace [AOR = 3.99; 95% CI: 1.38–6.83] were also significantly associated with PPE use.

Furthermore, workers with more than 3 years of experience had 1.85 times higher odds of PPE utilization compared to those with < 1 year [AOR = 1.85; 95% CI: 1.71–3.21]. Alcohol non-users were 3.13 times more likely to use PPE than alcohol users [AOR = 3.13; 95% CI: 2.18–4.57], while non-smokers were nearly twice as likely to utilize PPE compared to smokers [AOR = 1.97; 95% CI: 1.64–3.52] (Table 5). The value “1” indicates the reference category used in the regression analysis, serving as the baseline for comparison.

**Table 5:** Factors associated with the utilization of PPE among Workers in WA Oil factory, Debre Markos town, Ethiopia, 2024.

**Variable**	**Category**	**PPE utilization**		**COR (95% CI)**	**AOR (95%CI)**
		**Yes**	**No**		
Term of employment	Permanent	208	166	1	1
	Temporary	7	6	0.93 (0.73–0.98)	0.95 (0.76–0.99)
Safety training	Yes	111	31	3.98 (2.43–7.84)	4.77 (2.85–8.21)
	No	116	129	1	1
Work experience	< 1 year	68	67	1	
	1–3 years	85	72	1.25 (1.13–1.56)	1.34 (1.15–2.64)
	>3 years	61	34	1.76 (1.57–2.81)	1.85 (1.71–3.21)
PPE available	Yes	165	64	5.73 (2.47–9.02)	4.92 (2.35–7.12)
	No	49	109	1	1
Alcohol use	Yes	95	123	1	1
	No	120	49	3.17 (2.23–4.89)	3.13 (2.18–4.57)
Smoke cigarette	Yes	4	8	1	
	No	210	166	2.53 (1.91–4.10)	1.97 (1.64–3.52)
Health and safety supervision	Yes	140	99	1.38 (1.23–3.84)	2.81 (1.90–3.99)
	No	75	73	1	1
Safety Guidelines in the workplace	Yes	135	48	4.45 (2.92–7.06)	3.99 (1.38–6.83)
	No	79	125	1	1

COR, crude odds ratio; CI, confidence interval; AOR, adjusted odds ratio. Significant at a *P* < 0.05.

## Discussion

In this study, the magnitude of PPE utilization was 55.3%. This finding was higher than 38% in Addis Ababa (18), 35.43% and 41.7% in Debre Birhan studies in Ethiopia (2, 14), and 15.6% in Kampala, Uganda (10). However, this finding was lower than and 82.4% in Hawassa (22) studies in Ethiopia, 60% in Egypt (24), 86.4% in Nigeria (25), and 87.2% in Nawalparasi, Nepal (26). This finding is almost comparable with studies finding from the Kombolcha textile factory, Ethiopia, Adwa textile factory, Ethiopia (27), and Kampala, Uganda which indicated that 58.2%, 54.0%, and 50.4% of the workers had good PPE utilization, respectively (20, 28, 29). This disparity could be attributed to the differences in demographic information, study populations, workplace conditions, and employees' level of awareness about hazard control and disease prevention (12).

The findings of this study revealed that participation in safety training, the availability of PPE, consistent health and safety supervision, and the presence of workplace safety guidelines were significantly associated with higher levels of PPE utilization among workers. Specifically, individuals who had received safety training were nearly 5 times more likely to use PPE compared to those without such training. This result aligns with evidence reported in earlier studies, highlighting the importance of workplace training in promoting protective practices (18, 20, 27, 30, 31). Similarly, those who had not been trained on PPE utilization were less likely to utilize PPE in line with the previous studies (31, 32). This could be attributed to the fact that safety training helps reinforce compliance and encourages workers to adhere to safety protocols by fostering collaboration among employees, supervisors, and the factory's safety committee. Moreover, educating workers on proper PPE use not only enhances awareness but also plays a crucial role in promoting consistent and correct usage, ultimately contributing to the reduction of workplace injuries.

Consistent with findings from previous research (27, 28, 33, 34), this study found a strong association between supervision and PPE utilization. Workers who received supervision regarding PPE use were approximately three times more likely to use protective equipment compared to those who were not monitored. This may be because supervised employees are more likely to follow safety protocols due to reminders, accountability measures, or concerns about potential consequences such as warnings or disciplinary actions (35, 36). Furthermore, the study also highlighted that receiving safety orientation prior to starting work significantly influenced the likelihood of PPE use among workers.

The utilization of PPE was also significantly influenced by the presence of health and safety guidelines. Workers in areas where such guidelines were implemented were 4 times more likely to use PPE compared to those in workplaces without these guidelines. This finding aligns with a study conducted in Debre Berhan, Ethiopia, on PPE utilization among workers in large-scale factories (14). The establishment of health and safety guidelines likely encourages workers to properly adhere to PPE usage protocols, as the guidelines provide clear instructions and expectations for their use.

In this study, the two primary reasons for not using PPE were the unavailability of equipment (40.9%) and discomfort during use (24.6%). These findings are consistent with previous studies, which also cited the unavailability of PPE (26) and discomfort, particularly in extreme weather conditions, as significant barriers to its use (37, 38). This study is consistent with a similar study conducted in Nigeria (39), which identified discomfort and improperly sized PPE as key reasons for non-use. These issues may stem from several factors, including a lack of interest or awareness among workers, insufficient attention from responsible authorities, budget constraints, and the need for more comfortable and advanced PPE options.

Work experience was found to be a significant factor influencing PPE utilization among factory workers. This aligns with findings from studies in Kenya and Addis Ababa (18, 40), which observed that although employment type, income, and marital status did not significantly impact PPE use, work experience did play a role. A possible explanation is that workers with more years on the job are more likely to have undergone safety training and benefited from peer learning, making them more aware of workplace risks and the importance of using PPE consistently.

The current research found that the likelihood of using PPE was higher among workers who did not consume alcohol or smoke cigarettes compared to those who did. This may be due to the influence of substance use on workers' perceptions, which could lead to negligence in adhering to safety practices and an increased risk of work-related injuries. The association between substance use and PPE utilization was notable, with non-users being 3 times more likely to use PPE than users. This finding aligns with the study done in Hawassa (22), which suggested that individuals who use substances are less likely to engage in safety behaviors due to their tendency to take more risks. These findings underscore the need for greater focus on improving PPE usage by addressing the identified factors that influence its utilization. The study was cross-sectional, and we recommend using a more robust study design.

## Limitations of the study

Due to the cross-sectional design of the study, it was not possible to establish a cause-and-effect relationship between the predictor and outcome variables. Additionally, since the data were based on self-reports, there is a risk of response bias, as participants may have provided answers they perceived as socially desirable rather than accurately reflecting their actual behaviors.

## Conclusion

The utilization level of PPE among workers at the WA edible oil factory in Debre Markos Town was found to be moderate, emphasizing important gaps in occupational safety practices when compared to other developing countries. Key factors influencing PPE use included access to safety training, availability of equipment, workplace supervision, presence of safety guidelines, work experience, and lifestyle behaviors such as alcohol and tobacco use. To address these issues, it is recommended that the factory implement regular and practical safety training, ensure continuous availability of appropriate PPE, strengthen workplace supervision, and enforce clear safety guidelines. Additionally, promoting healthy lifestyle choices and discouraging substance use through workplace health programs can further enhance PPE compliance. These combined efforts are essential to improving worker safety and fostering a culture of prevention in industrial settings.

## Data Availability

The original contributions presented in the study are included in the article/supplementary material, further inquiries can be directed to the corresponding author.
